# Supplementary material for: New Insight into Assembled Fe3O4@PEI@Ag Structure as Acceptable Agent with Enzymatic and Photothermal Properties
Source: Int J Mol Sci. 2022 Sep 15;23(18):10743. doi: 10.3390/ijms231810743 (PMC9501236; doi:10.3390/ijms231810743)
Supplement: Supplementary file 1 [file ijms-23-10743-s001.zip › ijms-1807303-supplementary.pdf]

Supporting Information

New Insight into Assembled Fe_3O_4@PEI@Ag Structure as Acceptable Agent with Enzymatic and Photothermal Properties

Teng Wang[1,2#], Xi Hu[1,2#], Yujun Yang[3], Qing Wu[1,2], Chengdian He[1,2], Xiong He[1,2], Zhenyu Wang[1,2*], Xiang Mao[1,2*]

1. State Key Laboratory of Ultrasound in Medicine and Engineering, College of Biomedical Engineering, Chongqing Medical University, Chongqing, 400016, P. R. China.

2.Chongqing Key Laboratory of Biomedical Engineering, Chongqing Medical University, Chongqing, 400016, P. R. China.

3.Key Laboratory of Laboratory Medical Diagnostics, Ministry of Education, Department of Laboratory Medicine, Chongqing Medical Laboratory Microfluidics and SPRi Engineering Research Center, Chongqing Medical University, Chongqing 400016, P. R. China

Teng Wang and Xi Hu contributed equally in this work.

Supporting Information

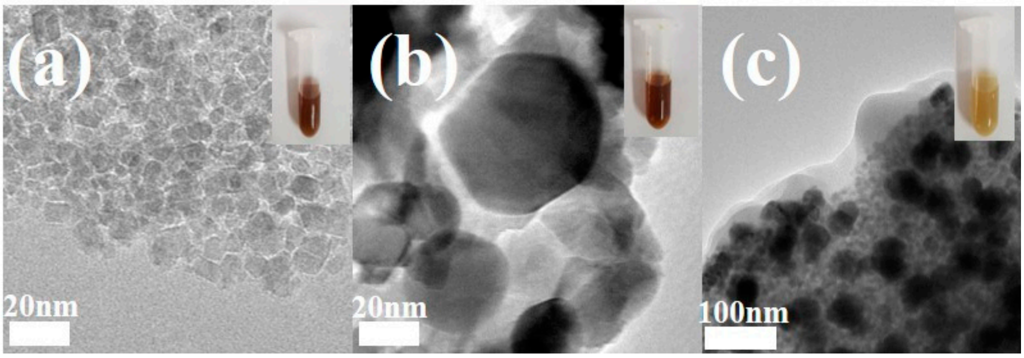

Figure S1 TEM imagines of (a) Fe_3O_4 NPs, (b) Ag NPs, (c) Fe_3O_4@PEI@Ag structure.

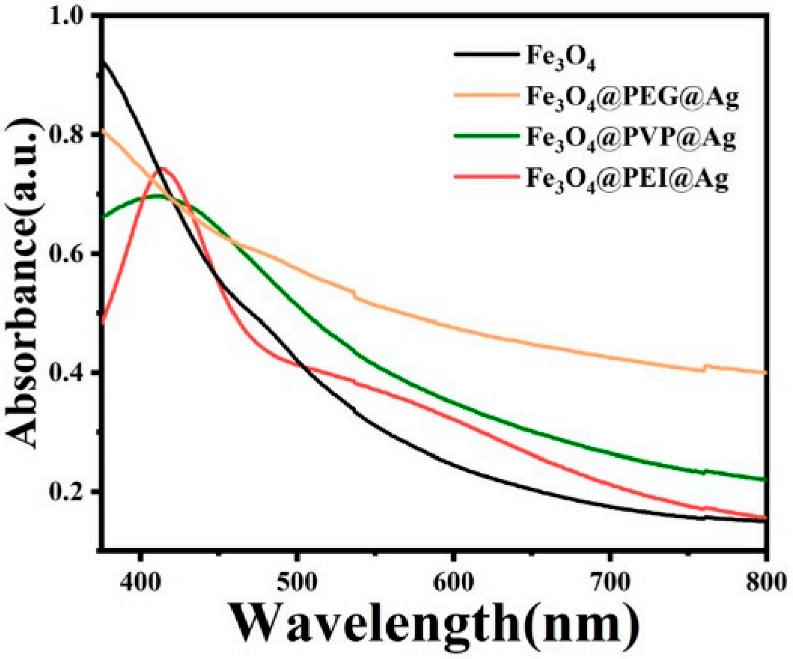

Figure S2 UV-vis absorbance spectra of Fe_3O_4 NPs, Fe_3O_4@PEG@Ag structure, Fe_3O_4@PVP@Ag structure and Fe_3O_4@PEI@Ag structure.

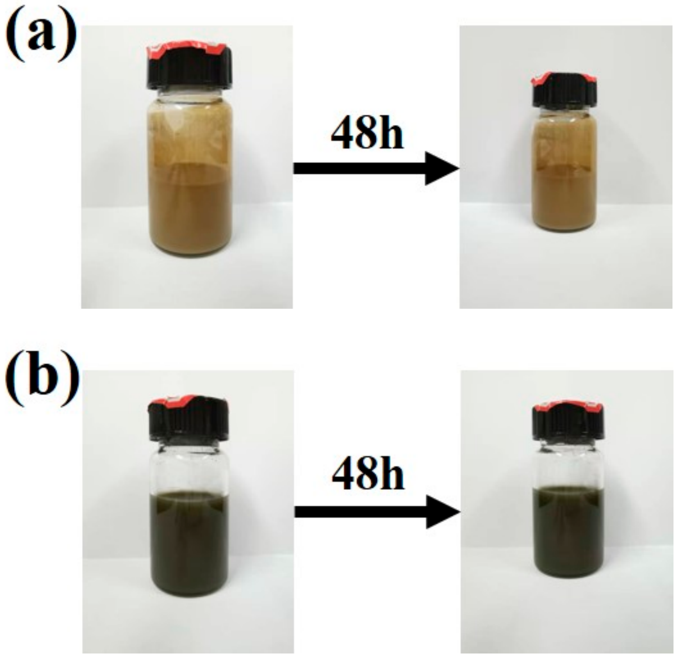

Figure S3 (a) The stability of Fe_3O_4@PEI@Ag structure after standing 48h; (b) The stability of Fe_3O_4@PEI@Ag@ICG structure after standing 48h

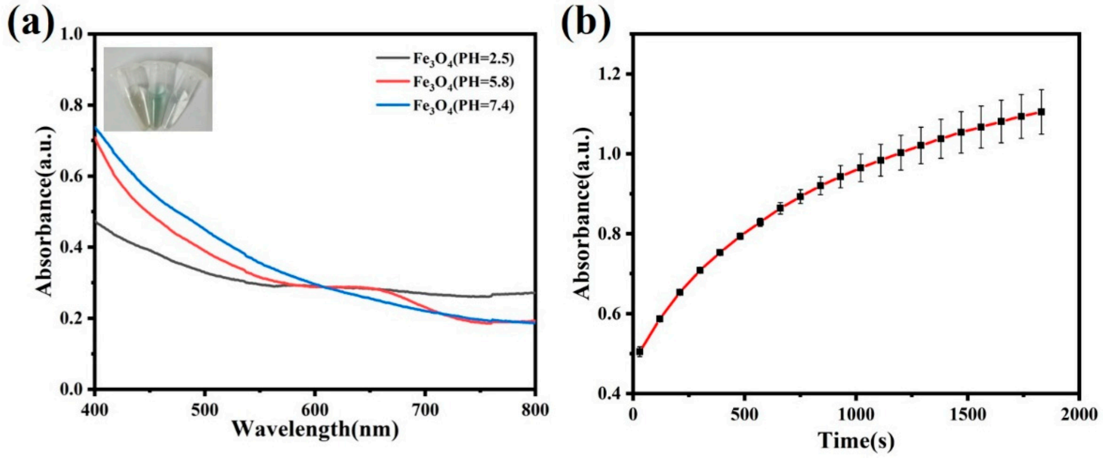

Figure S4 (a) Effect of pH on the peroxidase-like activity of Fe_3O_4 NPs; (b) Peroxidase-like activity of Fe_3O_4@PEI@Ag structure during 30 min.

Supporting Information

Table S1 Comparison of the kinetic data of Fe_3O_4@PEI@Ag structure with that of HRP in the previous literature

Nanozymes	K_m(mM)		V_{max}($\times10^{-7}$ M s^{-1})	
	H_2O_2	TMB	H_2O_2	TMB
Fe_3O_4@PEI@Ag structure	1.192	0.302	1.299	1.163
HRP[1]	3.7	0.434	0.87	1
$CiCo_2O_4$MS[2]	9.41	0.14	2.58	0.223
Ni-MOF nanosheet[3]	6.53	0.36	13	0.249
$ZnFe_2O_4$ MNPs[4]	1.66	0.85	0.774	1.331
Fe-Mil-88NH2[5]	2.06	0.028	1.047	0.704
Pd/Fe_3O_4-PEI-RGO[6]	3.66		0.4	
PdNPs[7]	0.21		0.021	

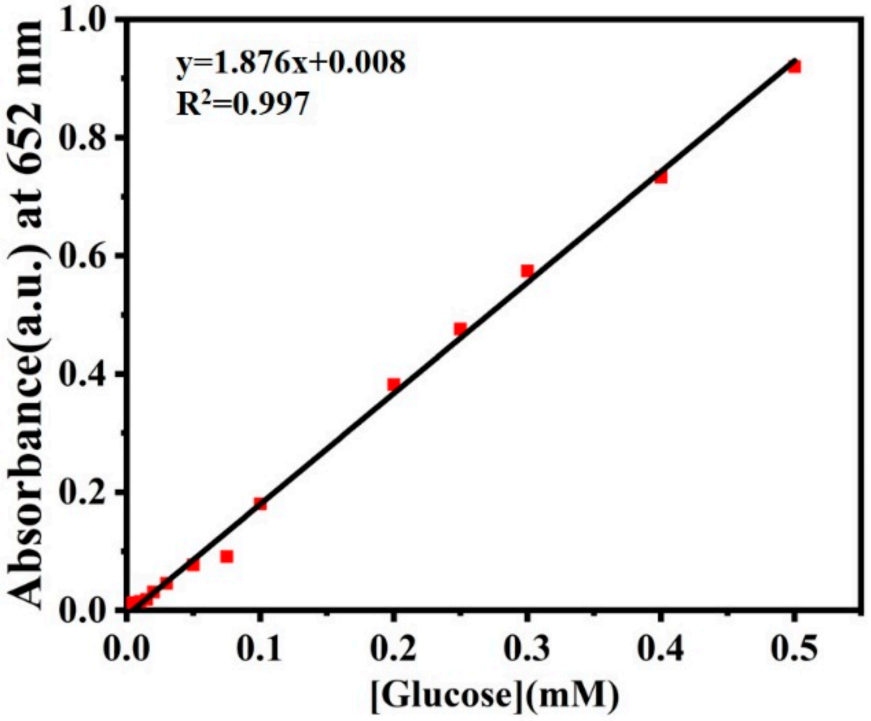

Figure S5 The linear relationship between the absorbance intensity and the concentration of glucose.

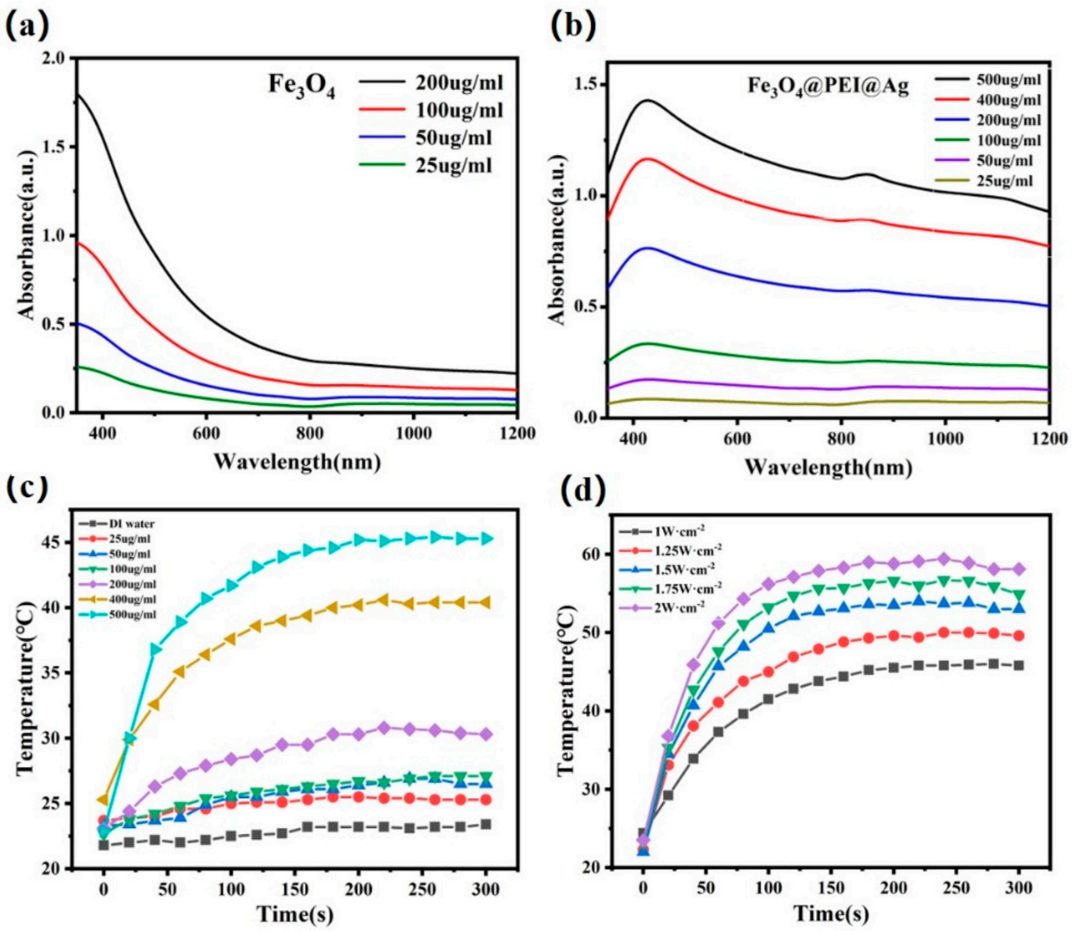

Figure S6 (a) Vis-NIR absorbance spectral of Fe_3O_4 NPs dispersions with different concentrations; (b) Vis-NIR absorbance spectral of Fe_3O_4@PEI@Ag structure dispersions with different concentrations; (c) Temperature increase of Fe_3O_4@PEI@Ag structure dispersion with gradient concentrations under 808 nm NIR laser irradiation; (d) Temperature increase of Fe_3O_4@PEI@Ag@ICG structure dispersion exhibited laser power density-dependent profiles.

References

1. Gao, L.; Zhuang, J.; Nie, L.; Zhang, J.; Zhang, Y.; Gu, N.; Wang, T.; Feng, J.; Yang, D.; Perrett, S.; Yan, X., Intrinsic peroxidase-like activity of ferromagnetic nanoparticles. *Nat Nanotechnol* **2007,** 2, (9), 577-83.
2. Huang, W.; Lin, T. Y.; Cao, Y.; Lai, X. Y.; Peng, J.; Tu, J. C., Hierarchical $NiCo_2O_4$ Hollow Sphere as a Peroxidase Mimetic for Colorimetric Detection of H_2O_2 and Glucose. *Sensors-Basel* **2017,** 17, (1).
3. Chen, J.; Shu, Y.; Li, H.; Xu, Q.; Hu, X., Nickel metal-organic framework 2D nanosheets with enhanced peroxidase nanozyme activity for colorimetric detection of H_2O_2. *Talanta* **2018,** 189, 254-261.
4. Su, L.; Feng, J.; Zhou, X.; Ren, C.; Li, H.; Chen, X., Colorimetric detection of urine glucose based $ZnFe_2O_4$ magnetic nanoparticles. *Anal Chem* **2012,** 84, (13), 5753-8.
5. Tan, H.; Li, Q.; Zhou, Z.; Ma, C.; Song, Y.; Xu, F.; Wang, L., A sensitive fluorescent assay for thiamine based on metal-organic frameworks with intrinsic peroxidase-like activity. *Anal Chim Acta* **2015,** 856, 90-5.
6. Li, S. L.; Li, H.; Chen, F. J.; Liu, J.; Zhang, H. L.; Yang, Z. Y.; Wang, B. D., Strong coupled palladium nanoparticles decorated on magnetic graphene nanosheets as enhanced peroxidase mimetics for colorimetric detection of H_2O_2. *Dyes Pigments* **2016,** 125, 64-71.
7. Kuo, C. H.; Lamontagne, L. K.; Brodsky, C. N.; Chou, L. Y.; Zhuang, J.; Sneed, B. T.; Sheehan, M. K.; Tsung, C. K., The effect of lattice strain on the catalytic properties of Pd nanocrystals. *ChemSusChem* **2013,** 6, (10), 1993-2000.
